# New annotations for three pea aphid genome assemblies allow comparative analyses of duplication and gene family evolution

**DOI:** 10.1093/g3journal/jkag136

**Published:** 2026-05-27

**Authors:** Kevin D Deem, Jennifer A Brisson

**Affiliations:** Department of Biology, University of Rochester, Rochester, NY 14627, United States; Department of Biology, University of Rochester, Rochester, NY 14627, United States

**Keywords:** gene annotation, genome assembly, gene family evolution, gene duplication, RNA-Seq, pea aphids

## Abstract

Reliable genome annotation is crucial for analyses of gene function, conservation, duplication, and evolution. Factors such as the sequencing technology used to create the assembly, as well as duplication and rearrangements within the genome of interest, can have a large impact on the quality of gene annotations. In particular, short-read-based assemblies tend to misassemble duplicated genes as single loci, a problem that requires additional measures such as long-read sequencing to resolve. Pea aphids exhibit a high level of gene duplication from frequent genomic rearrangements, which has led to the misassembly and misannotation of genes. Here, we re-annotate the pea aphid reference genome, along with 2 long-read pea aphid genomes, to facilitate future analyses of gene duplication and function in pea aphids. We use an integrated approach, consolidating both ab initio and RNA-Seq-based annotations into unified gene models. The new annotations contain genes that were missing, misannotated, or misassembled in the reference genome, and are generally consistent across assemblies, with the best agreement between the long-read assemblies. Our annotation method is sensitive enough to refine existing gene models, uncovering alternatively used promoters and isoforms, and aids in finding gene duplications. These data provide a useful supplement to the existing reference annotations and a new comparative framework for discovery and analysis of gene function and duplication in this important emerging model insect.

## Introduction

Proper assembly and annotation of genomes is crucial for bioinformatic and molecular biology investigations into gene function and evolution. As arguably one of the most important processes in gene family evolution, gene duplication presents a unique challenge for the proper assembly and annotation of genomes. The method(s) by which a genome assembly is produced have a substantial impact on the accuracy of gene duplicate assembly and annotation. One major deciding factor in accuracy of assembly is the choice of read type. Illumina short reads (50 to 300 bp) are readily obtained in high numbers in a cost-effective manner. However, short-read-based genome assemblies are the most prone to misassemble duplicated genes, especially for recent duplications (Alkan et al. 2011; Mastretta-Yanes et al. 2014; Wang et al. 2021). Long-read technologies such as Pacific Bioscience (Eid et al. 2009) or Oxford Nanopore Technologies (Jain et al. 2016), provide the most accurate assembly of overall gene structure, even in the trickiest cases of recent or partial gene duplication (Logsdon et al. 2020; Fadaie et al. 2021; Nguyen et al. 2024; Showpnil et al. 2024). Both methods of genome assembly can be improved to chromosome-level contiguity by various scaffolding methods, with chromosome conformation capture followed by high-throughput sequencing (Hi-C) being among the most modern and effective (Luo et al. 2021). Indeed, integration of short-, long-, and Hi-C-reads have produced some of the most accurate chromosome-level assemblies, even when highly repetitive sequences are present (Renschler et al. 2019; Miga et al. 2020).

Ab initio methods such as AUGUSTUS (Stanke and Morgenstern 2005) allow annotation of genes from raw genome sequence by predicting groups of exons that code for proteins. While these methods require no additional data beyond the assembled genome to perform gene annotation, the accuracy and completeness of the annotation suffers as a result. This accuracy can be increased by providing RNA-Seq data to better train models of splice sites using BRAKER1 (Hoff et al. 2016). Other RNA-Seq based methods of transcript discovery such as genome-guided Trinity (Grabherr et al. 2011) or StringTie (Pertea et al. 2015) use read sequence and/or coverage to identify exons and intron-spanning reads to determine how they are spliced into transcripts. These methods can be highly accurate and provide more complete gene models, but they can only identify genes expressed in RNA-Seq input (ie tissue-specific data will provide fewer genes than whole-body data). In addition, Trinity can also assemble transcriptomes de novo purely from RNA-Seq reads (the initial intended use of the software), potentially uncovering genes that are missing or misassembled in a given genome (Grabherr et al. 2011).

The software PASA takes a more unified approach, integrating Trinity de novo, Trinity genome-guided, BRAKER1, and StringTie transcript predictions into clustered gene models (Haas et al. 2008). These transcripts are first filtered for protein coding potential, quality of alignment to the genome, and intron size before being clustered into genes based on overlap (Haas et al. 2008). The advantage conferred by using PASA is that the complementary strengths of ab initio and RNA-Seq-based methods work synergistically when combined into a unified annotation. This integration of ab initio and RNA-Seq-based gene prediction is similar to the Eukaryotic Genome Annotation Pipeline tool that NCBI uses to automatically annotate reference genomes from publicly available data (Goldfarb et al. 2025).

Pea aphids (*Acyrthosiphon pisum*) are an important insect model for studying gene duplication (Fernández et al. 2020), the molecular genetic basis of morphological evolution (Li et al. 2020), and aphid-bacterial symbiont interactions (Price et al. 2011). However, these types of studies can be hindered by misassemblies within the pea aphid reference genome (Li et al. 2019). The current chromosome-level reference genome (v3) was generated through scaffolding of the previous assembly (v2; composed of Sanger and 454 reads) using Hi-C and the related Chicago technique (The International Aphid Genomics Consortium 2010; Putnam et al. 2016; Li et al. 2019). The authors of both assemblies found extensive repetitive sequences, horizontal gene transfers, and gene duplications (The International Aphid Genomics Consortium 2010; Li et al. 2019). Likely due to these factors, errors remain in the v3 assembly. For instance, long-read sequencing from winged and wingless males was required to properly assemble a region on the X chromosome controlling male wing dimorphism (Braendle et al. 2005; Li et al. 2020). This region was misassembled and largely absent in the v3 reference genome (Li et al. 2019), but the assembled long-read genome revealed the presence of a duplicated gene (*follistatin-3*) as part of an insertion specific to wingless males (Li et al. 2020). These assemblies, however, were never fully annotated outside of the male wing dimorphism locus, nor were the assembled sequences published (only reads). More recently, we investigated aphid-specific duplications of wing gene regulatory network components and found that a number of these newly identified paralogs were incorrectly annotated in the v3 reference genome (Saleh Ziabari et al. 2025a). These instances of misassembly and misannotation highlight the need for higher quality genome assemblies and annotations in this species.

One of the best ways to improve gene annotations is to use RNA-Seq data from a variety of morphs and life stages. Pea aphids alternate between live-bearing asexual females in the summer months, and sexual males and egg-laying females in the fall. They have 4 nymphal instars before becoming adults. In addition, the asexual females and sexual males have both winged and wingless morphs. The current pea aphid RefSeq annotation release, using the Eukaryotic Genome Annotation Pipeline, is composed of RNA-Seq data integrated with Gnomon (ab initio) gene predictions (Goldfarb et al. 2025). The RNA-Seq samples used to generate these annotations are mostly adults (51/59 datasets), with only a few cases of nymphs or embryos present, and no samples from later-stage embryos or from 1st or 2nd instar nymphs (https://www.ncbi.nlm.nih.gov/refseq/annotation_euk/Acyrthosiphon_pisum/103/). Further, information regarding the morph (winged vs wingless) is not available for most samples. Thus, these samples may not contain transcripts from genes or isoforms that are morph-specific.

Here our aim is to provide improved annotations of the reference assembly (v3, Li et al. 2019) corrected at the male wing dimorphism locus (Saleh Ziabari et al. 2025b), as well as the 2 assembled, but never fully annotated or published long-read assemblies. To do this, we combined previously generated RNA-Seq data from many morphs (asexual females, sexual females, males; winged and wingless when available) and developmental stages (embryos, nymphal instars, and adults; see Supplementary Fig. 1 for a full list of RNA-Seq sample types), along with our own newly-generated RNA-Seq data from an underrepresented stage and sample: winged and wingless male embryos. We used Trinity (genome-guided and de novo), StringTie, and BRAKER1 to individually predict transcripts, followed by PASA clustering of transcripts into unified gene models. Predicted proteins were then clustered with those of twenty other metazoan species using OrthoFinder to obtain functional annotation of gene models and quantify novel gene annotation. We have made our long-read assemblies, a version of the reference assembly with a corrected male wing dimorphism region, and their new annotations publicly available. These new gene annotations provide a rich source of information for future studies of duplication, function, and gene family evolution in pea aphids and other insects.

## Methods

A summary diagram of the samples used and the workflow described below is provided (Supplementary Fig. 1).

### Transcriptome data used for annotation

We used 22 previously generated RNA-Seq experiments totaling 58 readsets for our annotation efforts. 52 out of 58 of these readsets were not used in generation of the RefSeq annotation. Read accessions (Purandare et al. 2014; Grantham et al. 2020; Saleh Ziabari et al. 2025b) are provided in Supplementary Table 1.

We also generated new RNA-Seq data for young (stage 0 to 18) and old (stage 18 to 20) winged and wingless male embryos. Pea aphid mothers producing either winged or wingless males were generated as previously described (Saleh Ziabari et al. 2025b). Briefly, asexual females from lines producing exclusively winged (412) or wingless (409) males were reared in short day conditions to induce production of males and sexual females. Once mothers stop producing females, they begin producing only males, which was confirmed via PCR as in Saleh Ziabari et al. (2025b). Embryos were collected from the ovaries of male-producing mothers and sorted into young (germaria through onset of eye pigmentation) and old (onset of eye-pigmentation through birth) samples. Total RNA was extracted from embryos via TRIzol-chloroform extraction (Invitrogen, Carlsbad, CA) and treated with DNaseI. RNA clean-up was performed on 3ug of total RNA per replicate with the Zymo Clean & Concentrator-5 kit (Zymo Research, Irvine, CA). Three biological replicates of each of the 4 conditions (young and old embryos, wingless and winged of each) were then sequenced on an Illumina HiSeq 3000 producing 20 to 30 M 2 × 150 bp paired-end reads per replicate (Azenta Life Sciences, Burlington, MA). Raw reads were submitted to NCBI under BioProject PRJNA1380473.

### Genomes used in annotation

We used 3 genomes in this study. First, we used 2 long-read genomes, one generated from winged males and one from wingless males. These assemblies were generated by Li et al. (2020). We provide a brief description of how the sequences were assembled here (for further details, see Li et al. 2020). The winged assembly was produced using 14GB of Nanopore reads from winged males assembled with the MECAT (Xiao et al. 2017) software. The wingless assembly was made using 7GB of Nanopore reads from the wingless F2 line generated in Li et al. (2020), and 37GB of PacBio RSII reads from a different pea aphid line homozygous for the wingless insertion (LSR1, SRR15093657). These reads were assembled using MiniMap2 and Miniasm (Li 2016) using 4 rounds of Racon (Vaser et al. 2017) for the consensus step. Both winged and wingless long-read assemblies were polished with 10 rounds of Pilon (Walker et al. 2014) using >100X Illumina 2 × 100 bp short reads. We refer to these as the Winged (WD) Male Long-Read and Wingless (WL) Male Long-Read genomes. While the Nanopore reads generated by Li et al. (2020) were deposited to NCBI (winged: SRR 10028116, wingless: SRR8306868), the final assembled sequences were not published. As part of this study, we have deposited the final assembled sequences to NCBI under PRJNA1378148 (WD Male Long-Read) and PRJNA1378146 (WL Male Long-Read), while still citing the original Li et al. (2020) source within the Bioprojects.

Second, we used the reference v3 genome (Li et al. 2019), but altered to include an insertion that is present solely in wingless males (Li et al. 2020). Specifically, we soft-masked positions 62,565,000 to 62,660,000 on the X chromosome of that genome (Li et al. 2019) and added a new scaffold containing the properly assembled male wing dimorphism insertion region from the Wingless Long-Read assembly (Li et al. 2020) named “NC_WLinsert_pilon10_start62575000_to_62660000.” This Modified Reference, as we will refer to it here, was originally published without annotations in Saleh Ziabari et al. (2025b), but not deposited to NCBI. As part of this study, we have also deposited this Modified Reference assembly to NCBI under PRJNA1378164, citing both the source of the reference genome (Li et al. 2019) and the correction to the wingless allele (Saleh Ziabari et al 2025b) in the Bioprojects.

### Trinity de novo transcriptome assembly

Only paired-end reads were used to generate the Trinity de novo assembly (Grabherr et al. 2011). Reads were first adapter- and quality-trimmed with Trimmomatic v0.39 (Bolger et al. 2014) in paired-end mode using the parameters “ILLUMINACLIP: TruSeq3-PE-2.fa:2:30:10 SLIDINGWINDOW:4:5 LEADING:5 TRAILING:5 MINLEN:25.” Reads were then randomly subsampled to 2% using the view method in Sambamba v0.6.6 (Tarasov et al. 2015) with the parameter “-s 0.02.” Trinity (v2.15.1) de novo assembly of transcripts was then performed in singularity-ce v3.11.4 (Kurtzer et al. 2017) using the image file trinityrnaseq.v2.15.1.simg and the parameters “--normalize_by_read_set --no_parallel_norm_stats --min_kmer_cov 2.”

### Read mapping and RNA-Seq-based transcript assembly

Both single-end and paired-end samples (see Supplementary Table 1) were used for RNA-Seq-based gene annotation methods. Reads were aligned to each assembly with Hisat2 v2.2.1 (Kim et al. 2019) using default mapping parameters. The .sam files for each replicate from each sample were converted to .bam, sorted, merged, and indexed using SAMtools v1.13 (Li et al. 2009). Duplicate reads were removed from the merged replicate .bam files using the markdup function of Sambamba v0.6.6 with the parameter “--remove-duplicates.” These files were then each randomly subsampled at a rate of 20% using the view method of Sambamba with the parameter “-s 0.2.” These merged-replicate, duplicate-less, subsampled .bam files were then merged using SAMtools. Ultimately, this resulted in .bam files containing read counts of ∼300 M unique pair mappings for each assembly, a read count substantially exceeding the coverage typically required for transcript discovery. Braker was run in ET-mode (Hoff et al. 2016) on the respective processed .bam file for each assembly in singularity-ce v3.11.4 (Kurtzer et al. 2017) using the image braker3.sif (Gabriel et al. 2024). Genome-guided transcriptome assembly was performed with these files for each assembly in Trinity v2.15.1 (Grabherr et al. 2011), also run in singularity-ce v3.11.4 with the image trinityrnaseq.v2.15.1.simg, using the flag “--genome_guided_bam” and the parameter “--genome_guided_max_intron 200000.” Transcripts were mapped to the genome using GMAP v2021-08-25 (Wu and Watanabe 2005). StringTie v2.2.3 (Pertea et al. 2015) was run using default parameters and transcript sequences were extracted from genomic intervals using GffRead v0.12.7-2 (Pertea and Pertea 2020).

### Integrated gene clustering of transcripts using PASA

Transcript files from Trinity de novo (Trinity-DN) and genome-guided (Trinity-GG) assembly, and genomic coordinate files output from StringTie and Braker, were filtered and clustered into genes using PASA v2.5.3 (Haas et al. 2008) for all 3 assemblies. Following extraction of Trinity-DN accessions, clustering was run in singularity-ce v3.11.4 using the image file pasapipeline.v2.5.3.simg with the following parameters: “--create --run -I 100000 --ALIGNERS gmap --ALT_SPLICE –TRANSDECODER.” The final output included nucleotide sequences and genomic intervals for high-confidence protein-coding transcript isoforms with a maximum intron size of 100 kb clustered into “genes.”

### Protein prediction, BUSCO analysis, and ortholog clustering

Protein sequences coded by transcripts from Trinity-DN, and from Trinity-GG, StringTie → GffRead, Braker, and PASA for each genome assembly, were predicted separately using the “Longest-ORFs.fasta” output of TransDecoder (Haas n.d.) run either locally or on the Galaxy webserver (The Galaxy Community 2024). Gene-transcript maps output from Trinity-DN and Trinity-GG were provided using the “–gene_trans_map parameter.” For all transcript files, TransDecoder was run using the Universal Genetic Code with a minimum protein length of 100 amino acids “-m 100” in stranded mode “-S.”

Reference protein sequences were obtained from NCBI (O’Leary et al. 2016) from the RefSeq genomes for pea aphids and twenty other invertebrate and vertebrate species (*Aedes aegypti* [GCF_002204515.2], *Aphis gossypii* [GCF_020184175.1], *Apis mellifera* [GCF_003254395.2], *Acyrthosiphon pisum* [GCF_005508785.2], *Bombyx mori* [GCF_030269925.1], *Caenorhabditis elegans* [GCF_000002985.6], *Drosophila melanogaster* [GCF_000001215.4], *Daphnia pulex* [GCF_021134715.1], *Daktulosphaira vitifoliae* [GCF_025091365.1], *Homarus americanus* [GCF_018991925.1], *Homo sapiens* [GCF_000001405.40], *Homalodisca vitripennis* [GCF_021130785.1], *Mus musculus* [GCF_000001635.27], *Macrobrachium nipponense* [GCF_015104395.2], *Myzus persicae* [GCF_001856785.1], *Nilaparvata lugens* [GCF_014356525.2], *Neocloeon triangulifer* [GCF_031216515.1], *Rhopalosiphum maidis* [GCF_003676215.2], *Rhopalosiphum padi* [GCF_020882245.1], *Schistocerca gregaria* [GCF_023897955.1], and *Tribolium castaneum* [GCF_031307605.1]).

Protein files from gene annotation methods of the 3 assemblies of interest, Trinity-DN, and the RefSeq genomes above were used as input proteomes for ortholog clustering using OrthoFinder v2.5.5 (Emms and Kelly 2019). OrthoFinder clustered these proteins into orthogroups, which are groups of orthologs inferred to be descended from a single gene in the last common ancestor of all species present in the analysis. This initial OrthoFinder run, used for annotation purposes, included all isoforms for all genes. The raw clustering output from this run is located in Supplementary Table 3 and in the Dryad dataset as OrthoGroups.tsv. While this provided the most accurate annotations of isoforms, discrete clustering of isoforms can result in genes belonging to multiple orthogroups. Thus, a second run was performed that only included the proteins coded by the longest transcript of each gene, which ensured that each gene only belonged to a single orthogroup (gene family). The results of this second OrthoFinder run were then used for quantifying novel gene and gene family annotation (see sections on estimating gene number and evaluating novel gene and gene family annotation below).

BUSCO v5.2.2 (Manni et al. 2021; Tegenfeldt et al. 2025) was run on the TransDecoder Longest ORFs protein output for PASA, Trinity-GG, StringTie, and Braker for each assembly, as well as Trinity-DN, and the RefSeq proteins. OrthoDB version 10 protein databases for the Eukaryota, Metazoa, Arthropoda, and Insecta levels were used (Tegenfeldt et al. 2025). The results of this BUSCO analysis are available in Supplementary Table 4.

### Annotation of genomic interval files

The genomic intervals for mapped transcripts in PASA clusters for each genome assembly were annotated using the OrthoGroups.tsv file output from OrthoFinder, along with header information from the RefSeq protein fasta files for the twenty additional species obtained from NCBI, using custom python scripts. For each potential protein encoded in a transcript in an orthogroup, the first protein listed for a species within the same orthogroup had its header information appended to the corresponding transcript entry in the genomic intervals file as “Species_ortholog:header information.” In cases where a species had no proteins in that orthogroup, the ortholog is listed as “none” in the genomic intervals file. Annotated genomic interval files can be found in the PASA subfolder for each assembly in the Dryad dataset (See Data Availability Statement).

### Estimating gene number

The following was performed using custom python scripts. From the second OrthoFinder run, a new orthogroup table was generated replacing the protein identifiers for the pea aphid annotations with the gene locus identifiers (Supplementary Table 5). Orthogroups were then analyzed for the number of PASA clusters or RefSeq loci present for each pea aphid annotation. The total number of genes for each annotation was then summed based on presence in any orthogroup, or presence in an orthogroup containing: >1 pea aphid member, ≥1 non-pea aphid member, ≥1 non-aphid member, ≥10 non-pea aphid members, or ≥10 non-aphid members.

### Extracting novel annotations with *Drosophila* orthologs

Orthogroups containing PASA clusters for all 3 newly annotated assemblies, as well as ≥10 non-aphid members including *Drosophila melanogaster*, but no RefSeq pea aphid member, were extracted from the orthogroup table generated above for further examination using custom python scripts. Select annotations from this analysis are shown in Fig. 4.

### Evaluation of gene family representation

Orthogroup membership was again leveraged to assess the shared and unique representation of pea aphid gene families within the 4 annotations analyzed (RefSeq, Modified Reference, WL Male LR, WD Male LR). The orthogroup protein clusters output from the second OrthoFinder run comprise a bioinformatic proxy of gene families, which may contain zero, 1, or multiple genes from each pea aphid annotation. By counting the number of orthogroups containing genes from each assembly, we were able to assess the number of gene families present in 1, 2, 3, or all 4 pea aphid annotations (Fig. 6a). Importantly, this approach allowed us to solely analyze genes producing proteins greater than 100 amino acids in length that cluster based on sequence alignment.

### Quantification of shared and novel gene annotations

To quantify novel gene annotations, we examined the gene content for each annotation within groups of gene families that were present in:

1. All 4 annotations (RefSeq, Modified Reference, WL Male LR, WD Male LR)
2. Only the 3 new annotations (Modified Reference, WL Male LR, WD Male LR)
3. Only 2 of the new annotations
4. Only one of the new annotations

We counted genes within these gene families for each annotation for each of these conditions, as well single-copy genes, genes duplicated 2× to 9×, and genes duplicated ≥10× for each annotation within each group, as well as genes that were present as a single copy for all annotations within a group. The complete dataset and summary statistics are located in Supplementary Table 7.

### Visualization of results

Plots were generated from comma-separated value files output from custom python scripts visualized in Microsoft Office 365 Excel v2502 and edited in Adobe Photoshop v24.5.0. Gene diagrams were first visualized in Integrative Genomics Viewer v2.16.1 using annotated genomic interval files for PASA clusters in each assembly using a fixed window size across assemblies. Gene annotations were then isolated and compiled in Adobe Photoshop v24.5.0. The Venn diagram of orthogroup membership for each annotation was made using Interactivenn (Heberle et al. 2015).

## Results

**Fig. 1.**
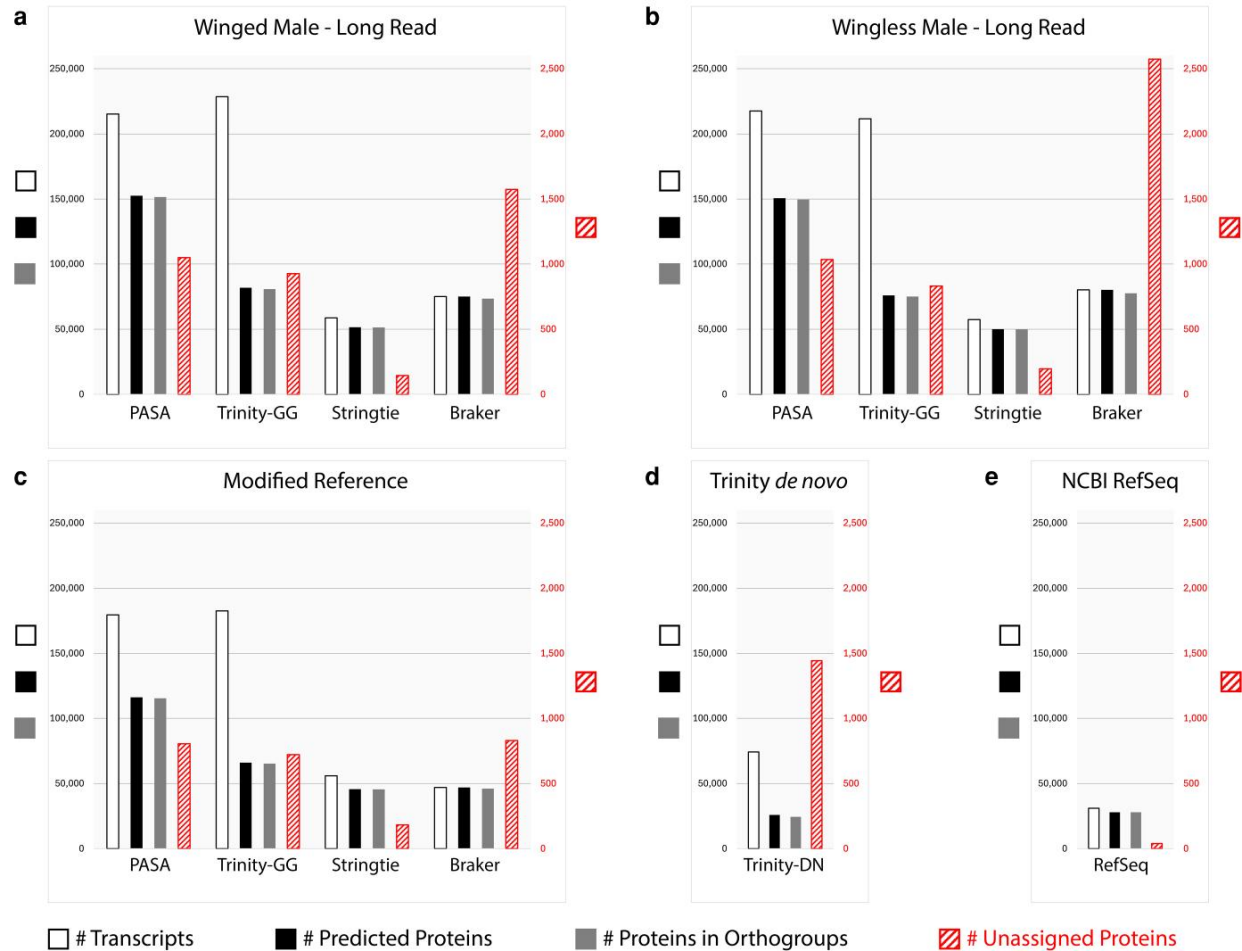
Relative amounts of coding and non-coding transcripts annotated by various methods. Number of predicted transcripts (white), protein-coding transcripts (black), and protein-coding transcripts in orthogroups (gray) are shown on the left axis. Number of predicted transcripts coding for proteins that were not assigned to any orthogroup (red hatched) are indicated on the right axis. All plots share the same scales for both axes. a to c) Numbers are shown for genome + RNA-Seq-based methods for each assembly. d) Numbers for Trinity de novo assembly of transcripts directly from RNA-Seq reads, integrated with predicted transcripts from the other 3 methods to produce PASA annotations for each assembly. e) Numbers for RefSeq annotations of transcripts and proteins in the reference genome.

The numbers of transcripts, predicted proteins, proteins in orthogroups, and unassigned proteins, for PASA, Trinity genome-guided, StringTie, and Braker for each assembly, as well as for Trinity de novo and pea aphid RefSeq annotations, are shown in Fig. 1. The raw data for this figure can be found in Supplementary Table 2. Integration of transcripts from the other 4 methods into PASA clusters consistently yielded the highest number of transcripts coding for proteins in orthogroups by clustering orthologs of 20 other species with OrthoFinder (Fig. 1a—to c; see Supplementary Table 3 for ortholog clustering). Braker consistently produced the highest percentage of unassigned proteins of the 4 genome + RNA-Seq-based methods (1.8%- to 3.2%) although Trinity de novo produced the highest percentage of unassigned proteins overall (5.6%, Fig. 1a to d, Supplementary Table 2). Trinity de novo also produced the lowest percentage of coding transcripts overall (34.8%), with Trinity genome-guided producing only slightly higher percentages of coding transcripts for each assembly (WD Male Long-Read: 35.8%, WL Male-Long Read: 35.9%, Modified Reference: 36.1%, Fig. 1a to c, Supplementary Table 2). Still, both Trinity methods produced a large number of total assembled transcripts relative to other methods, and thus still identified a high number of protein coding transcripts in orthogroups despite the low percentages (Fig. 1a to d, Supplementary Table 2). StringTie proved more conservative, consistently predicting much lower numbers of transcripts across each assembly (∼55.9 to 58.6 k) compared to Trinity genome guided, but with a much higher percentage of coding transcripts (81.6%- to 87.9%) and the lowest number and percentage of unassigned proteins of any of the methods (144 to 196, 0.3%- to 0.4%, Fig. 1a to c, Supplementary Table 2).

**Fig. 2.**
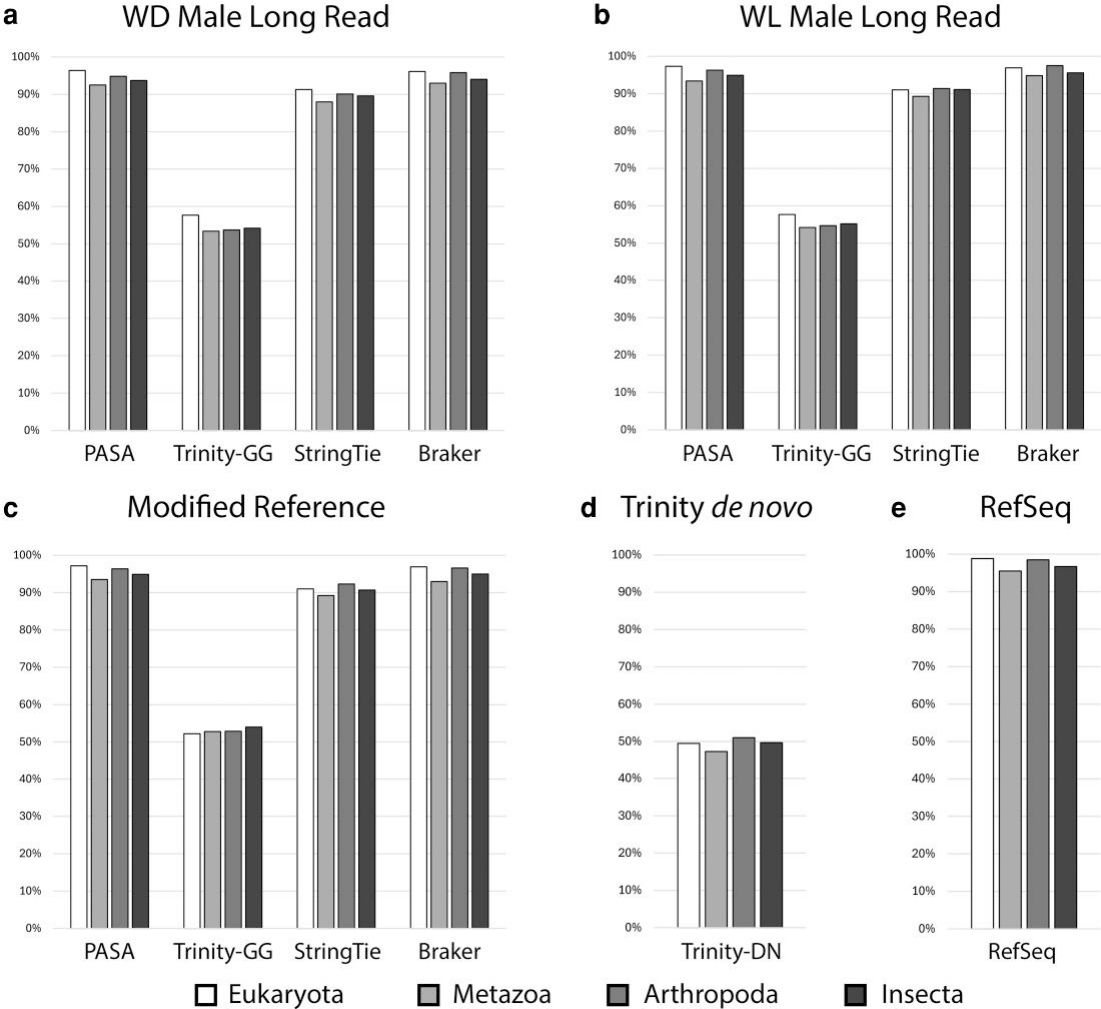
BUSCO analysis of proteomes. The percent of complete BUSCO's contained in PASA, Trinity genome-guided (Trinity-GG), StringTie, and Braker proteomes for the WD Male Long Read a), WL Male Long Read b), and Modified Reference c) assemblies, as well as Trinity de novo (Trinity-DN, d) and RefSeq proteomes e). Bar shade corresponds to the OrthoDB v10 dataset used: Eukaryota (white), Metazoa (light gray), Arthropoda (gray), or Insecta (dark gray).

To assess completeness of the proteomes produced by the various methods outlined above, we employed Benchmarking Universal Single-Copy Orthologs (BUSCO, Manni et al. 2021; Tegenfeldt et al. 2025). We evaluated proteome completeness for all methods, including the RefSeq annotations, at the Eukaryote, Metazoan, Arthropod, and Insect levels using version 10 databases from OrthoDB (Tegenfeldt et al. 2025). The results of this analysis are shown in Fig. 2 (see Supplementary Table 4 for exact percentages). Integration of transcripts from the 4 other methods into PASA clusters yielded highly complete predicted proteomes comparable to RefSeq (Fig. 2a to c and e). Focusing on the Insecta level, the methods that produced the most complete predicted proteomes were Braker (94.0%- to 95.6%) and PASA (93.7%- to 94.9%). The proteomes produced by those 2 methods were only slightly less complete than the RefSeq annotation (96.7%). This is despite Braker producing the highest percentages of unassigned proteins, likely through detection of Universal Single Copy Orthologs not expressed in our RNA-Seq dataset, which were then incorporated into PASA annotations. StringTie proteomes were less complete than those produced by Braker and PASA (89.6%- to 91.1%), but still considerably more complete than those produced by Trinity genome-guided (54.0%- to 55.2%) or Trinity de novo (49.7%).

Given widespread gene duplication known to occur in pea aphids (The International Aphid Genomics Consortium 2010; Li et al. 2019), we next asked how many genes are predicted within each of these genomes by our novel annotations. A recent gene number estimate, based on ESTs, cDNA libraries, and WQ-maker predictions, made during assembly of the chromosome-level reference genome, came to 40,090 (Li et al. 2019). We find that our annotation methods produced comparatively high numbers of PASA clusters (141,397 [WD Male LR], 145,815 [WL Male LR], 115,952 [Modified Reference]), likely containing pseudogenes, transposons, and potentially, endosymbiont contamination.

**Fig. 3.**
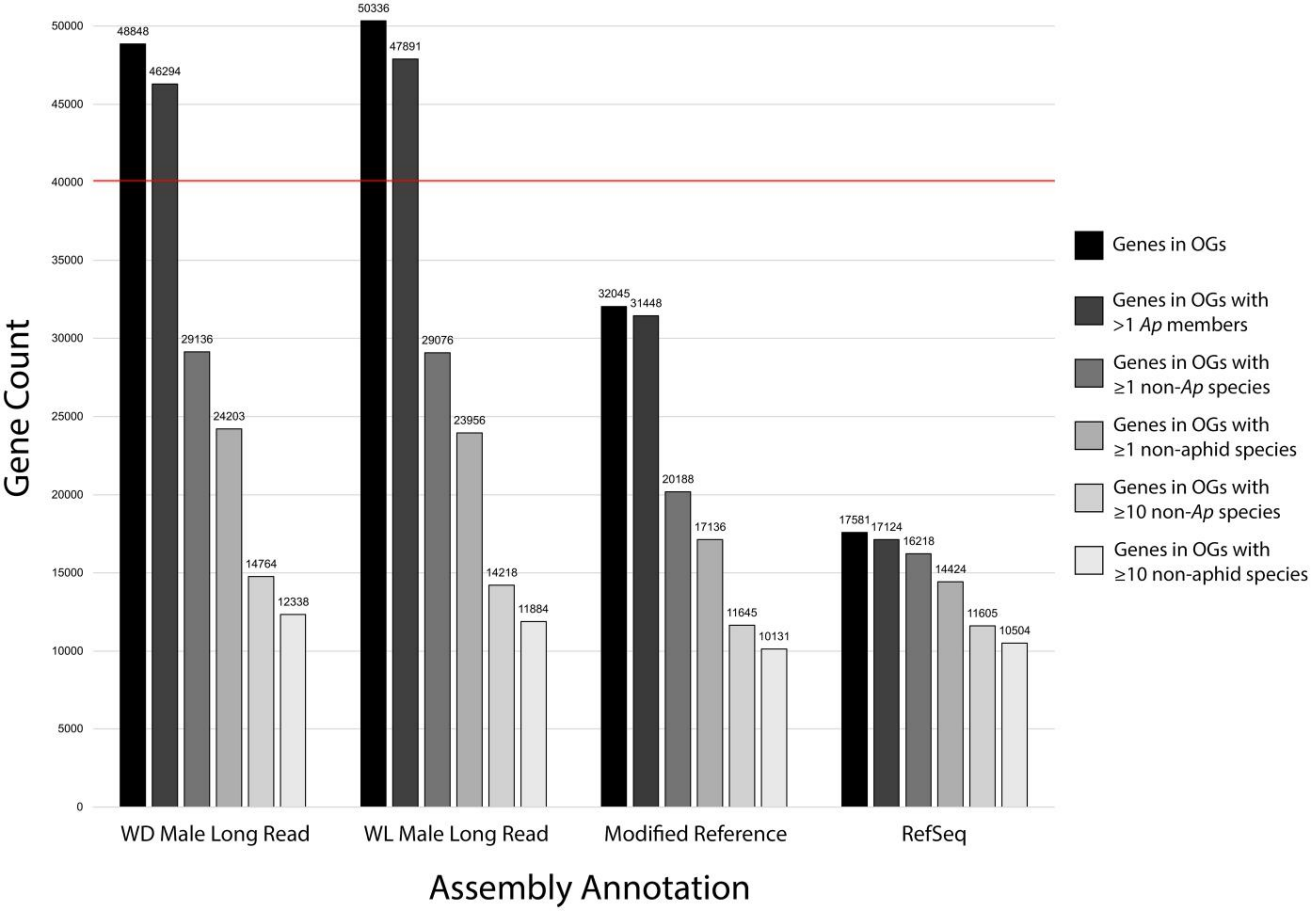
Gene count estimates in pea aphid assemblies with orthologs of varying evolutionary distance. Numbers of PASA gene clusters for each assembly, or RefSeq genes, producing protein-coding transcripts assigned to orthogroups containing members of varying evolutionary distance. The categories are as follows: all genes in orthogroups, genes in orthogroups with at least one other member from another pea aphid assembly, at least 1 non-pea aphid species, at least 1 non-aphid species, at least 10 non-pea aphid species, or at least 10 non-aphid species. The number of genes for each category is indicated at the top of the bar. The red line indicates the 40,090 gene estimate from the most recent genome assembly (Li et al. 2019).

While our first run of OrthoFinder with all transcripts provided the most accurate gene annotation, orthogroups composed of specific isoforms can complicate analyses of gene duplication. We reran OrthoFinder with only protein sequences from the longest transcription units (isoform covering the most genomic distance) for each gene. This ensured that the orthogroups comprised bona fide gene families, and that no genes belonged to multiple orthogroups. The results of this clustering are in Supplementary Table 5. We then assessed more stringent gene estimates based on the number of PASA clusters (genes) that code for a protein in an orthogroup with members from other pea aphid assemblies and/or twenty other eukaryotic species (Fig. 3). Requiring that a PASA cluster from a given assembly codes for a protein in an orthogroup with at least one other protein from another pea aphid assembly produced gene count estimates more similar to the 40,090 estimate of Li et al. 2019 (46,294 [WD Male LR], 47,891 [WL Male LR], 31,448 [Modified Reference]) (Fig. 3, Supplementary Table 6). The number of genes in orthogroups drops sharply as requirements on the number of orthogroup members from more distantly related species increases (Fig. 3), suggesting that lineage-specific gene duplicates are rapidly diverging.

**Fig. 4.**
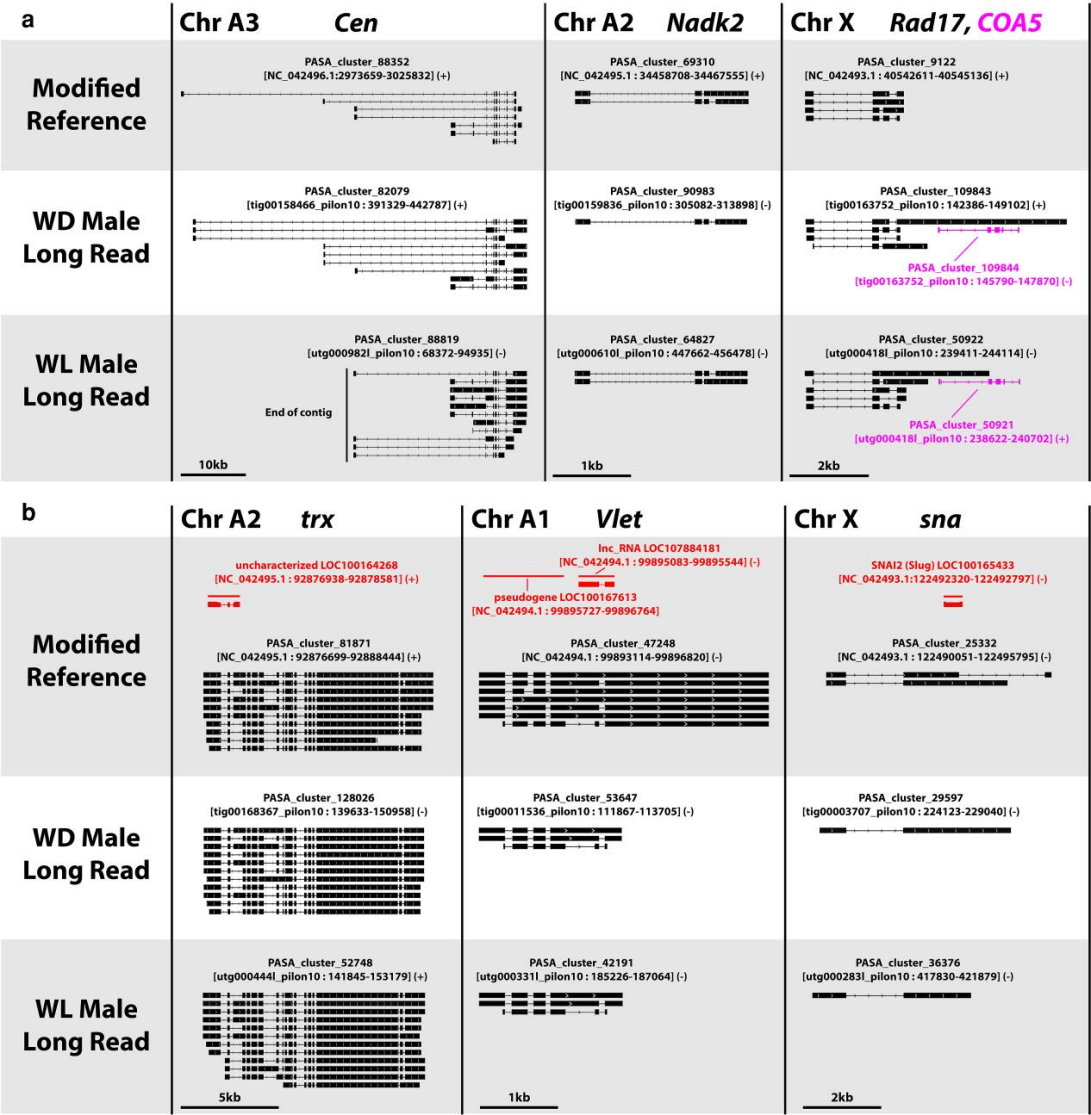
PASA assemblies of missing and misannotated genes. Gene structure diagrams for PASA-assembled genes that are either missing or incorrectly annotated in the RefSeq features. RefSeq annotations are shown in red, while newly annotated PASA clusters in the Modified Reference, WD Male Long Read, and WL Male Long Read assemblies are shown in black or magenta. The chromosomal location within the Modified Reference genome is indicated next to the gene name(s). Genes are shown at different scales, indicated by scale bars at the bottom. For clarity, overlapping annotations of other genes are not shown. a) PASA-assembled gene structures for the genes *Centrocortin* (*Cen*), *Nad-kinase 2* (*Nadk2*), *Rad17 checkpoint clamp loader component* (*Rad17*) (all black), and *Cytochrome-c oxidase assembly factor 5* (*COA5*, magenta) that are absent from the RefSeq annotation. The edge of the contig adjacent to the WL Male Long Read annotation of *Cen* is marked by a black line. b) PASA assemblies for the genes *trithorax* (*trx*), *Valette* (*Vlet*), and *snail* (*sna*) that are incorrectly annotated in the RefSeq features.

While a large number of newly annotated genes appear to be lineage-specific, there are also ∼1,200 more genes with 10 or more non-aphid orthologs in the long-read genomes vs the short-read reference (Fig. 3, Supplementary Table 6). We searched for genes in orthogroups with no RefSeq member but 10 or more non-aphid members, including *Drosophila melanogaster*, to identify newly annotated pea aphid orthologs of conserved fly genes. Using this method, we found genes that are missing from the RefSeq annotations (Fig. 4a), as well as genes whose misannotation or misassembly disrupted protein clustering (Fig. 4b). These include missing annotations for *Centrocortin* (*Cen*), *Nad-kinase 2* (*Nadk2*), *Rad17 checkpoint clamp loader component* (*Rad17*), *Cytochrome-c oxidase assembly factor 5* (*COA5*) (Fig. 4a), and misannotation of the genes *trithorax* (*trx*), *Valette* (*Vlet*), and *snail* (*sna*) (Fig. 4b).

**Fig. 5.**
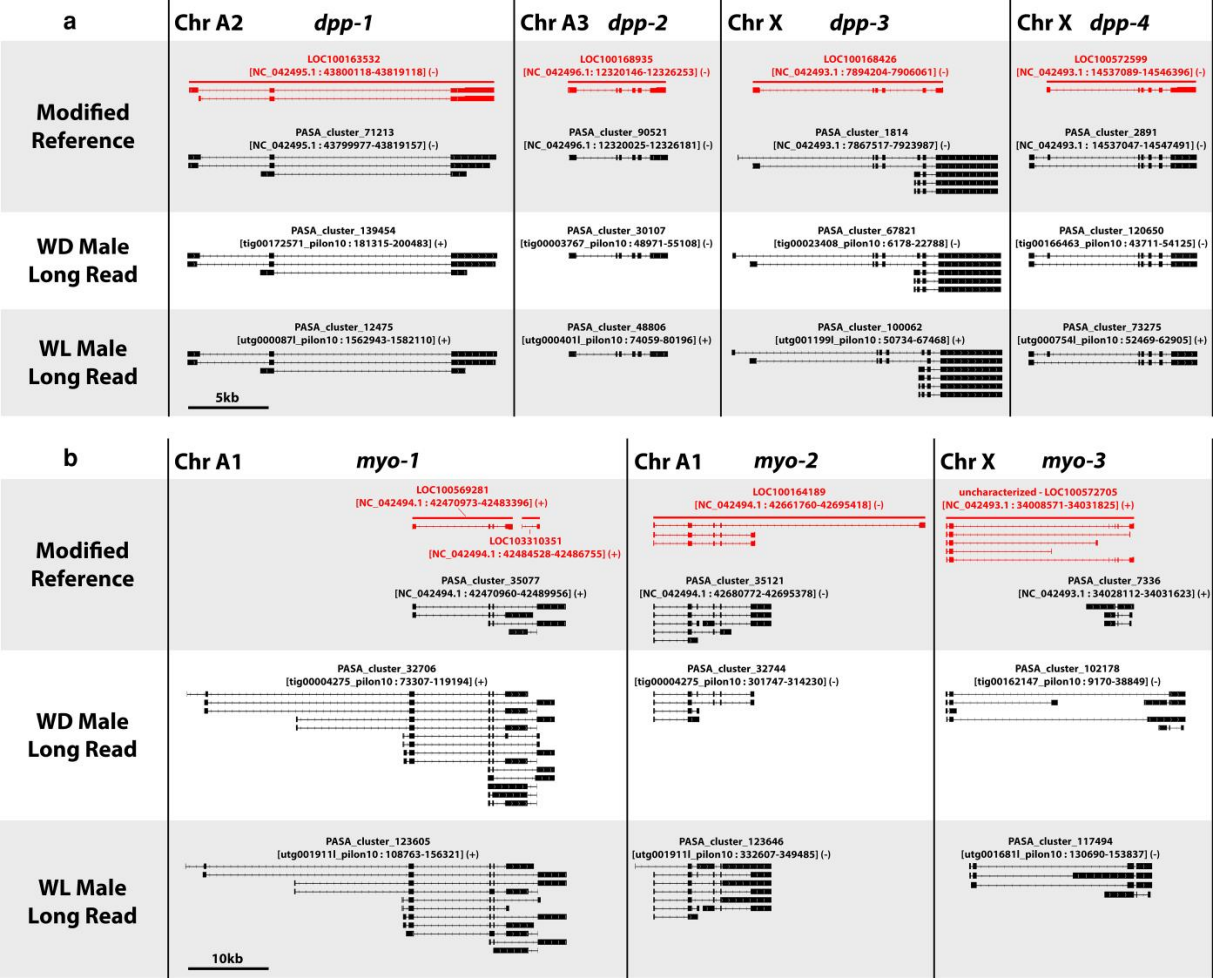
Annotations of duplicated TGF-β signaling ligands. Gene structure diagrams for paralogs of the TGF-β signaling ligands *decapentaplegic* (*dpp*) a) and *myoglianin* (*myo*) b) across pea aphid genome assemblies. RefSeq annotations are shown in red, PASA annotations are shown in black. For clarity, overlapping annotations of other genes are not shown. a) Annotations for all 4 *dpp* paralogs, shown at the same scale. Scale bar: 5 kb b) Annotations for the 3 paralogs of the gene *myo*, shown at the same scale. Scale bar: 10 kb.

Based on our ability to detect and correct previously unannotated and misannotated genes, we checked if these data support the previous identification of 4 paralogs of the BMP-branch TGF-β ligand *dpp* (Brisson et al. 2010; Saleh Ziabari et al. 2025a). We were able to confirm the presence and gene structure of all 4 paralogs across all 3 assemblies (Fig. 5a). Predicted gene structures, however, did differ between RefSeq features (red) and PASA clusters (black). Our PASA clusters contain novel isoforms using alternative promoters for *dpp-1* and *dpp-3* and predict that the promoter for *dpp-4* is upstream of the RefSeq annotations (Fig. 5a). These refined promoter annotations for the *dpp* paralogs are consistent across all 3 genome assemblies.

Recent findings have shown that paralogs of the gene *follistatin* are important for pea aphid wing dimorphisms (Li et al. 2020; Saleh Ziabari et al. 2025b). While we have speculated that morph-specific transcripts from *dpp* duplicates may play a role in these dimorphisms (Saleh Ziabari et al. 2025a), potentially interacting with *follistatin* duplicates, Dpp is not known to interact with Follistatin *in vivo* (Bickel et al. 2008; Pentek et al. 2009). For this reason, we set out to identify potential gene duplication for the Activin branch TGF-β ligand and primary Follistatin target, *myoglianin* (Bickel et al. 2008; Pentek et al. 2009). We identified 3 paralogs of *myoglianin* present in all 3 assemblies (Fig. 5b). Two fragmented RefSeq annotations for *myo-1* are actually a single gene with a large duplicated exon containing much of the coding sequence for the separate protein isoforms. PASA clusters in the Long-Read assemblies agree on additional upstream promoters for *myo-1* that are absent from the RefSeq features and PASA clusters in the Modified Reference, indicating potential misassembly of this region in the reference (Fig. 5b). The RefSeq annotation of *myo-2* indicates an alternative downstream exon not supported by RNA-Seq-based annotation in any of the 3 genomes (Fig. 5b). *myo-3* is a pseudogene, with different intact ORFs present in each of the 3 genome assemblies, responsible for differences in ortholog clustering between them (Fig. 5b). That is, PASA clusters from the Modified Reference and WD Male Long-Read assemblies code for proteins that cluster with other *myoglianin* paralogs and orthologs, but the RefSeq annotation and the PASA cluster in the WL Male Long-Read assembly do not.

**Fig. 6.**
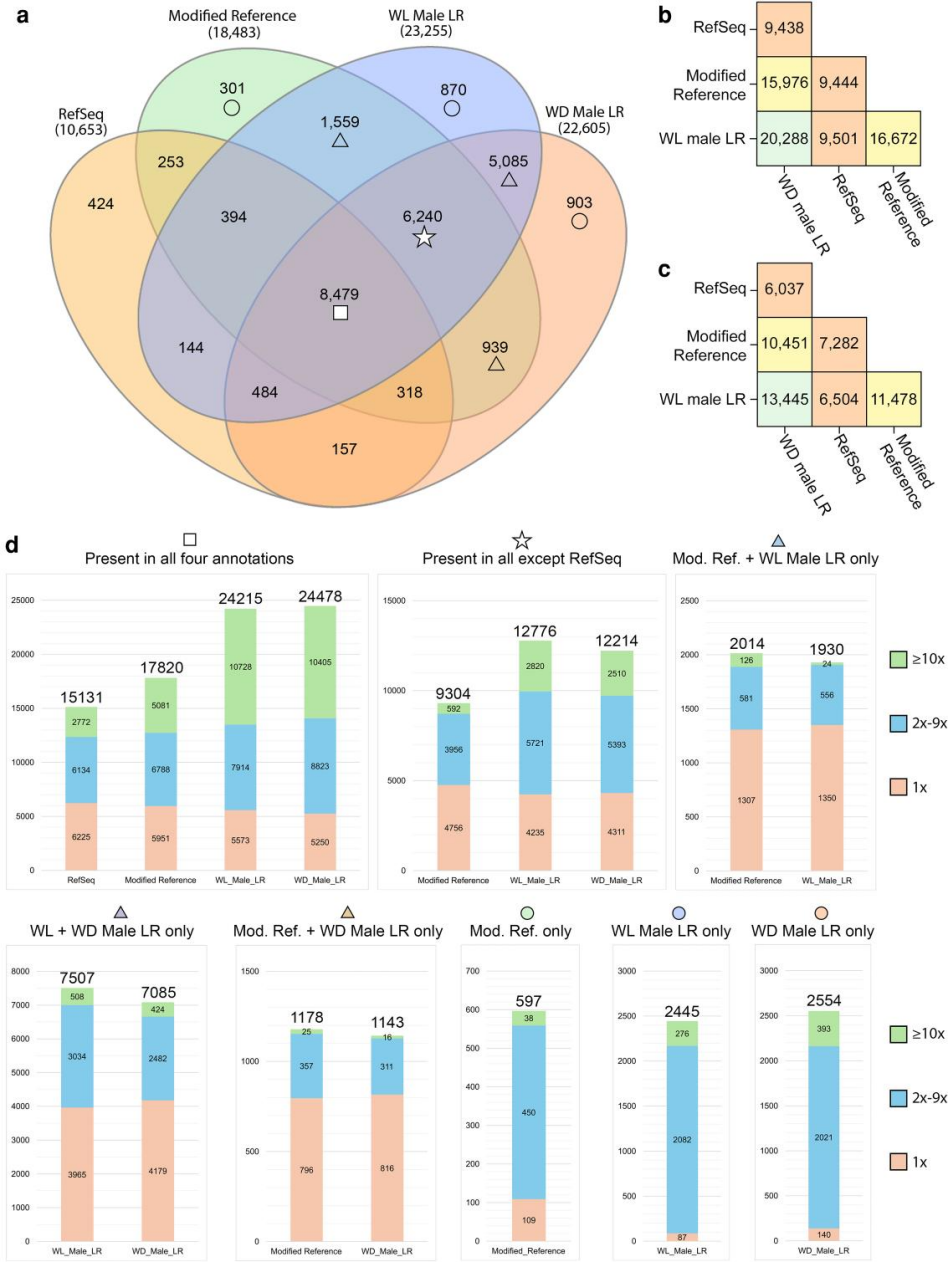
Newly annotated genes and gene families. Genes and gene families present in the RefSeq and new gene annotations. a) Overlap of gene families present in each gene annotation. The total number of gene families present in each annotation is indicated under the name. Gene families present in all 4 annotations are indicated by a square. Those present in all 3 new annotations, but not the RefSeq, are indicated by a star. Those present in 2 out of the 3 new annotations but not the RefSeq are marked by triangles. Gene families that are uniquely present in a single new annotation are indicated by a circle. b and c) Pairwise agreement between assemblies for the presence of genes in the same gene families. The best agreement, found between new annotations of the WD and WL Long-Read assemblies, is indicated by green shading. The next best agreement, found between the new annotations of the Modified Reference assembly and either the WD or WL Long-Read assembly, is indicated by yellow shading. The worst agreement, found between the RefSeq annotation and the 3 new annotations, is indicated by orange shading. b) Number of orthogroups containing at least 1 gene from both annotations in each pairwise comparison. c) Number of orthogroups containing the same copy number of genes from both annotations in each pairwise comparison. d) Graphs of gene content for each annotation within the groups of gene families indicated by shapes in a). Genes are separated into categories based on duplication level: either single-copy (orange), duplicated between 2 and 9 times (blue), or duplicated 10 or more times (green). The number of genes falling into each category for each annotation in each group of gene families is indicated in the corresponding shaded region of the bar. The total number of genes present in each annotation for that group of gene families is shown at the top of the bar.

We evaluated how many previously annotated and newly annotated genes and gene families are present in our new annotations. First, we asked how many pea aphid gene families that are present in the original RefSeq annotation are also present in our new annotations. Of the 10,653 gene families in the RefSeq annotation, 10,229 (96.0%) were present in at least one of our new annotations, while only 424 (4.0%) were not found in any of the new annotations. Further, 8,479 (79.6%) of these were present in all 3 of our new annotations (Fig. 6a, square). Next, we asked how many previously unannotated gene families are in our new annotations. 6,240 gene families were present in all 3 new annotations (Fig. 6a, star), but not the RefSeq. Clustering of the proteins from these genes from all 3 assemblies suggests that they were properly assembled in the reference genome, but were missed in the RefSeq annotation, likely due to the low diversity of RNA-Seq samples used. An additional 7,583 gene families were present in 2 out of the 3 new annotations, but not RefSeq (Fig. 6a, triangles). Of those gene families present in 2 out of 3 assemblies, the majority (5,085) were only shared between our WD and WL Long-Read assemblies. These likely contain gene families whose members are misassembled in the reference genome, and thus only appear in the long-read assemblies. In total 13,823 newly annotated pea aphid gene families were found, present in at least 2 out of 3 of our new annotations, but missing from the RefSeq annotation.

We performed pairwise comparisons between our new annotations and RefSeq to assess agreement in gene family content. We split this analysis between simple agreement, having at least 1 gene in the same gene families (Fig. 6b), and strict agreement, having the same number of genes in the same gene families (Fig. 6c). The best agreement in both was found between annotations of the WD and WL Long-Read assemblies, each having genes in 20,288 of the same gene families, with 13,445 of those (66.3%) containing the same number of genes from both annotations (Fig. 6b and c, green shading). The second-best agreement was found in comparisons of our new annotation of the Modified Reference assembly and the annotations of our WD and WL Long-Read assemblies. The Modified Reference annotation contained genes in 15,976 to 16,672 of the same gene families as the long-read annotations, with 10,451 to 11,478 having the same number of genes (Fig. 6b and c, yellow shading). The worst agreement was found in comparisons of the RefSeq annotation with our new annotations, with RefSeq only having genes in 9,438 to 9,501 of the same gene families, and with only 6,037 to 7,282 of those having the same number of genes (Fig. 6b and c, orange shading). To quantify the number of novel gene annotations within the newly annotated gene families, we counted the genes within gene families present in all 4 annotations (RefSeq, Modified Reference, WL Male LR, WD Male LR), or present in 1 or more of the new annotations, but not RefSeq. The full dataset along with summary statistics can be found in Supplementary Table 7.

We used the 8,479 gene families represented in all 4 annotations to assess how the numbers of genes within those families varied across annotations. Within these gene families, the RefSeq annotation contained a total of 15,131 genes, while our annotation of the Modified Reference genome contained 17,820, a difference of 2,689 genes. Since the WL male-specific inserted sequence that was corrected in the Modified Reference assembly only contains a handful of genes (Li et al. 2020), the vast majority of those genes are likely gene duplicates missing in the RefSeq annotation. Further, the WL and WD Long-Read annotations contain 24,215 and 24,478 genes within these gene families, which is 6,395 and 6,658 more genes than the Modified Reference annotation, respectively. Thus, ∼6,500 gene duplicates are likely misassembled in the reference genome, just within the 8,479 gene families present in all 4 annotations. Notably, the majority of these duplicates exist in copy numbers greater than 10× (Fig. 6d, square), suggesting that they consist of mobile elements.

We consider genes in the newly annotated gene families but not present in RefSeq to fall into 3 categories: either high-, medium-, or low-confidence, based presence in 3/3, 2/3, or 1/3 of our new annotations. The fewest genes make up the-low confidence category (Modified Reference: 597, WL Male Long-Read: 2,445, WD Male Long-Read: 2,554), with considerably more in the medium-confidence category (Modified Reference: 3,192, WL Male Long-Read: 9,015, WD Male Long-Read: 8,228), and the most in the high-confidence category (Modified Reference: 9,304, WL Male Long-Read: 12,776, WD Male Long-Read: 12,214). Importantly, the duplication levels within the low, medium, and high-confidence genes in newly annotated gene families are similar to that of the RefSeq annotation (specifically that relatively few genes are duplicated >10×, compare Fig. 6d, star, triangles, and circles to RefSeq in Fig. 6d square), suggesting that these are not transposons, but rather, many are aphid-specific, and particularly pea-aphid-specific, genes and paralogs, as suggested by the evolutionary analysis in Fig. 3.

Of particular interest are the 5,085 newly annotated gene families only found in the 2 long-read assemblies. This specificity to annotations of the long-read assemblies suggests that their component genes are also misassembled in the reference assembly. However, unlike the misassembled gene duplicates in the 8,479 gene families present in all 4 annotations, detected in ≥10× copy number in the long-read assemblies (likely mobile elements, Fig. 6a and d, square), the long-read assembly-specific gene family members exist in relatively low copy number (Fig. 6a and d, purple triangle). Of the 7,507 (WL Male Long-Read) and 7,085 (WD Male Long-Read) genes in the 5,085 long-read-specific gene families, 3,965 (52.8%) and 4,179 (59.0%) are single-copy, 3,034 (40.4%) and 2,482 (35.0%) are duplicated 2× to 9×, and only 506 (6.7%) and 424 (6.0%) are duplicated ≥10×, respectively. Thus, these comprise ∼7,000 newly annotated low-copy number genes present in ∼5,000 gene families that were likely misassembled in the reference genome.

## Discussion

Here we present new gene annotations leveraging RNA-Seq data from across life stages, morphs, and sex, for 3 pea aphid genome assemblies. We integrated annotations from multiple methods (Trinity de novo, Trinity genome-guided, StringTie, Braker-ET) into PASA gene clusters, maximizing the number of proteins in orthogroups (Fig. 1) and overall completeness as determined by BUSCO (Fig. 2).

Gene count estimates from our annotations of the long-read (46,294/47,891) and short-read (31,448) genomes lie above and below, respectively, the previous estimate of 40,090 for the reference genome (Fig. 3, Supplementary Table 6) (Li et al. 2019). For the long-read genomes, >20 k of these genes are predicted to be lineage-specific, while only half as many lineage-specific genes are annotated in the short-read reference (Fig. 3). This large disparity in gene count is not observed for more broadly conserved genes. New annotations on all 3 assemblies and RefSeq agree on 10,504 to 12,338 genes which cluster with orthologs in at least 10 non-aphid species (Fig. 3, Supplementary Table 6). There are likely a number of factors contributing to this large disparity in count estimates for new genes.

The difference in pea aphid gene counts for the reference genome (31,448 in this study, 40,090 in Li et al. 2019) may stem from the numerous differences in estimation methods, as well as our requirement that proteins cluster with those from another pea aphid assembly or the RefSeq proteins. One likely major contributor is that most of the 40,090 predicted genes from Li et al. (2019) do not have associated RefSeq annotations or reference proteins. Additionally, for rapidly evolving gene duplicates that are not part of the reference protein set, misassembly in the short-read reference genome may lead to predicted protein sequences that do not cluster with proteins from the properly assembled loci in the long-read genomes. Thus, these misassembled genes would not show up in our gene count estimate. Further, even when misassembly of duplicates into a single locus in the reference does not disrupt clustering with properly assembled paralogs in the long-read genomes, the gene count estimate for the reference only increases by 1, while it increases by more than 1 for the long-read assemblies.

The 2 long-read genomes (WD and WL Male) were derived from closely related, inbred lines. Thus, when paralogs are properly assembled in 1 long-read genome, it is more likely that they will be similarly assembled, annotated and clustered, keeping gene estimates similar. However, there is also the possibility that the gene counts in the long-read genomes (46,294/47,891) are artificially inflated by redundant contig fragments. Given these factors, we find it reasonable that we obtained gene counts above and below the 40,090 predicted by Li et al. (2019) for our long-read and reference genomes, respectively. Still, further analyses are required to determine where the true gene count lies within the range shown in Fig. 3.

The new annotations provided by integrating multiple methods into PASA gene clusters have identified previously missing or misannotated genes in the reference assembly (Fig. 4). These annotations have also helped in confirming previous reports on *dpp* gene duplication (Fig. 5a) as well as identified a previously unknown *myo* duplicate pseudogene (Fig. 5b). The new annotations also identify novel promoters for many of these gene duplicates, and consistency of the gene models across the 3 assemblies strongly suggests that they are accurate (Fig. 5).

Differences were observed between the assemblies in the annotated gene structures and protein clustering for the *myo3* pseudogene (Fig. 5b). This seems to be due to differential fragmentation of the ancestral ORF by different premature stop codons in each assembly, disrupting transcript discovery, gene structure annotation, and protein clustering. This highlights an additional challenge in initial pseudogene annotation and detection with a genome assembly generated from a single genetic line. However, such disagreement in structure and coding potential across assemblies may be a hallmark of pseudogenes. Detection of such discrepancies requires a comparative approach, which the data presented here help to facilitate for pea aphids.

Ultimately, we detected many newly annotated genes that fall into several categories. First, within the 8,479 gene families present in all 4 annotations, we detected 2,689 (Modified Reference), 9,084 (WL Male Long-Read), and 9,347 (WD Male Long-Read) new genes, most of which are duplicated ≥10× and likely newly annotated transposon insertions (Fig. 6a and d, square). Second, the 8,738 gene families found in the Modified Reference and at least one of the long-read genomes, but not the RefSeq annotation, comprise an additional 12,496 genes (in the Modified Reference annotation). This brings the total for newly annotated genes present in the reference assembly, but missing in the RefSeq annotation, due to lower RNA-Seq sample diversity, to 15,185 (∼3,000 of which are suspected transposon copies). Finally, the 5,085 gene families found within both long-read assemblies, but neither annotation of the reference assembly, comprise an additional 7,085 to 7,507 genes of mostly low-copy number that were most likely misassembled in the reference genome.

These data provide a useful supplement to existing RefSeq annotations by identifying and correcting missing and misannotated genes. The annotations of the 2 long-read genomes provide a platform for investigating misassembly of multiple loci into a single locus in the short-read reference genome. These annotations will aid in future investigations into the structure, function, and evolution of genes and gene families.

## Supplementary Material

jkag136_Supplementary_Data

## Data Availability

The resources produced in this work have been made available through deposition to public repositories as well as inclusion in the Supplementary data files. The genome assemblies annotated in this work have been deposited to NCBI under BioProjects PRJNA1378164 (Modified Reference), PRJNA1378146 (Wingless Male Long-read), and PRJNA1378148 (Winged Male Long-Read). The male embryo RNA-Seq reads generated in this study were deposited under BioProject PRJNA1380473. Additionally, the predicted transcripts, proteins, and genomic coordinates produced by all methods for all 3 assemblies, annotated genomic coordinate files for PASA genes, genome sequences with original chromosome names, and the ortholog clustering information for all predicted proteins and those from 20 other species have been deposited to Dryad (https://doi.org/10.5061/dryad.s1rn8pknd). Supplemental material available at G3 online.

## References

[jkag136-B1] Alkan C, Sajjadian S, Eichler EE. 2011. Limitations of next-generation genome sequence assembly. Nat Methods. 8:61–65. 10.1038/nmeth.1527. 21102452 PMC3115693

[jkag136-B2] Bickel D, Shah R, Gesualdi SC, Haerry TE. 2008. Drosophila follistatin exhibits unique structural modifications and interacts with several TGF-β family members. Mech Dev. 125:117–129. 10.1016/j.mod.2007.09.013. 18077144 PMC2278114

[jkag136-B3] Bolger AM, Lohse M, Usadel B. 2014. Trimmomatic: a flexible trimmer for illumina sequence data. Bioinformatics. 30:2114–2120. 10.1093/bioinformatics/btu170. 24695404 PMC4103590

[jkag136-B4] Braendle C, Caillaud MC, Stern DL. 2005. Genetic mapping of *aphicarus* – a sex-linked locus controlling a wing polymorphism in the pea aphid (*Acyrthosiphon pisum*). Heredity (Edinb). 94:435–442. 10.1038/sj.hdy.6800633. 15674387

[jkag136-B5] Brisson JA, Ishikawa A, Miura T. 2010. Wing development genes of the pea aphid and differential gene expression between winged and unwinged morphs. Insect Mol Biol. 19:63–73. 10.1111/j.1365-2583.2009.00935.x. 20482640

[jkag136-B6] Eid J et al 2009. Real-time DNA sequencing from single polymerase molecules. Science. 323:133–138. 10.1126/science.1162986. 19023044

[jkag136-B7] Emms DM, Kelly S. 2019. OrthoFinder: phylogenetic orthology inference for comparative genomics. Genome Biol. 20:238. 10.1186/s13059-019-1832-y. 31727128 PMC6857279

[jkag136-B8] Fadaie Z et al 2021. Long-read technologies identify a hidden inverted duplication in a family with choroideremia. Hum. Genet. Genomics Adv. 2:100046. 10.1016/j.xhgg.2021.100046. PMC8756506 35047838

[jkag136-B9] Fernández R et al 2020. Selection following gene duplication shapes recent genome evolution in the pea aphid *Acyrthosiphon pisum*. Mol Biol Evol. 37:2601–2615. 10.1093/molbev/msaa110. 32359152 PMC7475028

[jkag136-B10] Gabriel L et al 2024. BRAKER3: fully automated genome annotation using RNA-seq and protein evidence with GeneMark-ETP, AUGUSTUS, and TSEBRA. Genome Res. 34:769–777. 10.1101/gr.278090.123. 38866550 PMC11216308

[jkag136-B11] Goldfarb T et al 2025. NCBI RefSeq: reference sequence standards through 25 years of curation and annotation. Nucleic Acids Res. 53:D243–D257. 10.1093/nar/gkae1038. 39526381 PMC11701664

[jkag136-B12] Grabherr MG et al 2011. Full-length transcriptome assembly from RNA-Seq data without a reference genome. Nat Biotechnol. 29:644–652. 10.1038/nbt.1883. 21572440 PMC3571712

[jkag136-B13] Grantham ME, Shingleton AW, Dudley E, Brisson JA. 2020. Expression profiling of winged- and wingless-destined pea aphid embryos implicates insulin/insulin growth factor signaling in morph differences. Evol Dev. 22:257–268. 10.1111/ede.12326. 31682317 PMC7196481

[jkag136-B14] Haas BJ , n.d. https://github.com/TransDecoder/TransDecoder.

[jkag136-B15] Haas BJ et al 2008. Automated eukaryotic gene structure annotation using EVidenceModeler and the program to assemble spliced alignments. Genome Biol. 9:R7. 10.1186/gb-2008-9-1-r7. 18190707 PMC2395244

[jkag136-B16] Heberle H, Meirelles GV, Da Silva FR, Telles GP, Minghim R. 2015. InteractiVenn: a web-based tool for the analysis of sets through Venn diagrams. BMC Bioinformatics. 16:169. 10.1186/s12859-015-0611-3. 25994840 PMC4455604

[jkag136-B17] Hoff KJ, Lange S, Lomsadze A, Borodovsky M, Stanke M. 2016. BRAKER1: unsupervised RNA-seq-based genome annotation with GeneMark-ET and AUGUSTUS. Bioinformatics. 32:767–769. 10.1093/bioinformatics/btv661. 26559507 PMC6078167

[jkag136-B18] Jain M, Olsen HE, Paten B, Akeson M. 2016. The Oxford Nanopore MinION: delivery of nanopore sequencing to the genomics community. Genome Biol. 17:239. 10.1186/s13059-016-1103-0. 27887629 PMC5124260

[jkag136-B19] Kim D, Paggi JM, Park C, Bennett C, Salzberg SL. 2019. Graph-based genome alignment and genotyping with HISAT2 and HISAT-genotype. Nat Biotechnol. 37:907–915. 10.1038/s41587-019-0201-4. 31375807 PMC7605509

[jkag136-B20] Kurtzer GM, Sochat V, Bauer MW. 2017. Singularity: scientific containers for mobility of compute. PLoS One. 12:. 10.1371/journal.pone.0177459. PMC5426675 28494014

[jkag136-B21] Li B et al 2020. A large genomic insertion containing a duplicated follistatin gene is linked to the pea aphid male wing dimorphism. eLife. 9:e50608. 10.7554/eLife.50608. 32141813 PMC7060042

[jkag136-B22] Li H et al 2009. The sequence alignment/map format and SAMtools. Bioinformatics. 25:2078–2079. 10.1093/bioinformatics/btp352. 19505943 PMC2723002

[jkag136-B23] Li H . 2016. Minimap and miniasm: fast mapping and de novo assembly for noisy long sequences. Bioinformatics. 32:2103–2110. 10.1093/bioinformatics/btw152. 27153593 PMC4937194

[jkag136-B24] Li Y, Park H, Smith TE, Moran NA. 2019. Gene family evolution in the pea aphid based on chromosome-level genome assembly. Mol Biol Evol. 36:2143–2156. 10.1093/molbev/msz138. 31173104 PMC6759078

[jkag136-B25] Logsdon GA, Vollger MR, Eichler EE. 2020. Long-read human genome sequencing and its applications. Nat Rev Genet. 21:597–614. 10.1038/s41576-020-0236-x. 32504078 PMC7877196

[jkag136-B26] Luo J et al 2021. A comprehensive review of scaffolding methods in genome assembly. Brief Bioinform. 22:bbab033. 10.1093/bib/bbab033. 33634311

[jkag136-B27] Manni M, Berkeley MR, Seppey M, Simão FA, Zdobnov EM. 2021. BUSCO update: novel and streamlined workflows along with broader and deeper phylogenetic coverage for scoring of eukaryotic, prokaryotic, and viral genomes. Mol Biol Evol. 38:4647–4654. 10.1093/molbev/msab199. 34320186 PMC8476166

[jkag136-B28] Mastretta-Yanes A et al 2014. Gene duplication, population genomics, and species-level differentiation within a tropical mountain shrub. Genome Biol Evol. 6:2611–2624. 10.1093/gbe/evu205. 25223767 PMC4224332

[jkag136-B29] Miga KH et al 2020. Telomere-to-telomere assembly of a complete human X chromosome. Nature. 585:79–84. 10.1038/s41586-020-2547-7. 32663838 PMC7484160

[jkag136-B30] Nguyen AK, Blacksmith MS, Kidd JM. 2024. Duplications and retrogenes are numerous and widespread in modern canine genomic assemblies. Genome Biol Evol. 16:1–19. 10.1093/gbe/evae142. PMC11259980 38946312

[jkag136-B31] O’Leary NA et al 2016. Reference sequence (RefSeq) database at NCBI: current status, taxonomic expansion, and functional annotation. Nucleic Acids Res. 44:D733–D745. 10.1093/nar/gkv1189. 26553804 PMC4702849

[jkag136-B32] Pentek J, Parker L, Wu A, Arora K. 2009. Follistatin preferentially antagonizes activin rather than BMP signaling in *Drosophila*. Genesis. 47:261–273. 10.1002/dvg.20486. 19241394

[jkag136-B33] Pertea GM, Pertea M. 2020. GFF utilities: GffRead and GffCompare. F1000Res. 9:304. 10.12688/f1000research.23297.2. PMC7222033 32489650

[jkag136-B34] Pertea M et al 2015. StringTie enables improved reconstruction of a transcriptome from RNA-seq reads. Nat Biotechnol. 33:290–295. 10.1038/nbt.3122. 25690850 PMC4643835

[jkag136-B35] Price DRG, Duncan RP, Shigenobu S, Wilson ACC. 2011. Genome expansion and differential expression of amino acid transporters at the aphid/Buchnera symbiotic interface. Mol Biol Evol. 28:3113–3126. 10.1093/molbev/msr140. 21613235

[jkag136-B36] Purandare SR, Bickel RD, Jaquiery J, Rispe C, Brisson JA. 2014. Accelerated evolution of morph-biased genes in pea aphids. Mol Biol Evol. 31:2073–2083. 10.1093/molbev/msu149. 24770714 PMC4104313

[jkag136-B37] Putnam NH et al 2016. Chromosome-scale shotgun assembly using an in vitro method for long-range linkage. Genome Res. 26:342–350. 10.1101/gr.193474.115. 26848124 PMC4772016

[jkag136-B38] Renschler G et al 2019. Hi-C guided assemblies reveal conserved regulatory topologies on X and autosomes despite extensive genome shuffling. Genes Dev. 33:1591–1612. 10.1101/gad.328971.119. 31601616 PMC6824461

[jkag136-B39] Saleh Ziabari O, Deem KD, Zhong Q, Brisson JA. 2025a. Analysis of duplication and potential functional divergence of wing gene network components in pea aphids. Evol Dev. 27:e70021. 10.1111/ede.70021. 41212073

[jkag136-B40] Saleh Ziabari O et al 2025b. Gene duplication captures morph-specific promoter usage in the evolution of aphid wing dimorphisms. Proc Natl Acad Sci U S A. 122:e2420893122. 10.1073/pnas.2420893122.

[jkag136-B41] Showpnil IA et al 2024. Long-read genome sequencing resolves complex genomic rearrangements in rare genetic syndromes. Npj Genomic Med. 9:66. 10.1038/s41525-024-00454-4. PMC11655636 39695126

[jkag136-B42] Stanke M, Morgenstern B. 2005. AUGUSTUS: a web server for gene prediction in eukaryotes that allows user-defined constraints. Nucleic Acids Res. 33:W465–W467. 10.1093/nar/gki458. 15980513 PMC1160219

[jkag136-B43] Tarasov A, Vilella AJ, Cuppen E, Nijman IJ, Prins P. 2015. Sambamba: fast processing of NGS alignment formats. Bioinformatics. 31:2032–2034. 10.1093/bioinformatics/btv098. 25697820 PMC4765878

[jkag136-B44] Tegenfeldt F et al 2025. OrthoDB and BUSCO update: annotation of orthologs with wider sampling of genomes. Nucleic Acids Res. 53:D516–D522. 10.1093/nar/gkae987. 39535043 PMC11701741

[jkag136-B45] The Galaxy Community . 2024. The Galaxy platform for accessible, reproducible, and collaborative data analyses: 2024 update. Nucleic Acids Res. 52:W83–W94. 10.1093/nar/gkae410. 38769056 PMC11223835

[jkag136-B46] The International Aphid Genomics Consortium . 2010. Genome sequence of the pea aphid *Acyrthosiphon pisum*. PLoS Biol. 8:e1000313. 10.1371/journal.pbio.1000313. 20186266 PMC2826372

[jkag136-B47] Vaser R, Sović I, Nagarajan N, Šikić M. 2017. Fast and accurate de novo genome assembly from long uncorrected reads. Genome Res. 27:737–746. 10.1101/gr.214270.116. 28100585 PMC5411768

[jkag136-B48] Walker BJ et al 2014. Pilon: an integrated tool for comprehensive microbial variant detection and genome assembly improvement. PLoS One. 9:e112963. 10.1371/journal.pone.0112963. 25409509 PMC4237348

[jkag136-B49] Wang P, Meng F, Moore BM, Shiu S-H. 2021. Impact of short-read sequencing on the misassembly of a plant genome. BMC Genomics. 22:99. 10.1186/s12864-021-07397-5. 33530937 PMC7852129

[jkag136-B50] Wu TD, Watanabe CK. 2005. GMAP: a genomic mapping and alignment program for mRNA and EST sequences. Bioinformatics. 21:1859–1875. 10.1093/bioinformatics/bti310. 15728110

[jkag136-B51] Xiao C-L et al 2017. MECAT: fast mapping, error correction, and de novo assembly for single-molecule sequencing reads. Nat Methods. 14:1072–1074. 10.1038/nmeth.4432. 28945707

